# Long-Term Study of the Effects of Environment, Variety, and Fertilisation on Yield and Stability of Spring Barley Grain

**DOI:** 10.3390/plants13192745

**Published:** 2024-09-30

**Authors:** Lukáš Hlisnikovský, Veronika Zemanová, Muhammad Roman, Ladislav Menšík, Eva Kunzová

**Affiliations:** 1 Department of Nutrition Management, Crop Research Institute, Drnovská 507, Ruzyně, 161 01 Prague, Czech Republic; veronika.zemanova@vurv.cz (V.Z.); ladislav.mensik@vurv.cz (L.M.); kunzova@vurv.cz (E.K.); 2 Department of Environment, Faculty of Environment, Jan Evangelista Purkyně University, Pasteurova 15, 400 96 Ústí nad Labem, Czech Republic; m.maan26@outlook.com

**Keywords:** *Hordeum vulgare* L., climate change, weather pattern, grain production, nitrogen optimisation, inter-annual yield variability

## Abstract

The stability and yield of barley grain are affected by several factors, such as climatic conditions, fertilisation, and the different barley varieties. In a long-term experiment in Prague, Czech Republic, established in 1955, we analysed the weather trends and how weather, fertilisation (10 treatments in total), and different barley varieties affected grain yield and stability. A total of 44 seasons were evaluated. Trends in mean, minimum, and maximum temperatures from 1953 to 2023, as well as sunshine duration from 1961 to 2022, showed statistically significant increases. The trend for annual precipitation from 1953 to 2023 was not significant, but changes in precipitation were recorded via seasonal precipitation concentration indexes. The unfertilised Control and farmyard manure (FYM) provided the lowest mean yields. Mineral fertilisers (NPK) and FYM+NPK increased grain yield, ranging from 4.9 t ha^−1^ to 5.5 t ha^−1^. Three notable correlations between weather conditions and yields were observed: (1) June precipitation (r = 0.4), (2) minimal temperature in July (r = 0.3), and (3) sunshine duration in May (r = −0.5). According to the linear–plateau response model, the reasonable N dose is 55 kg ha^−1^, resulting in a mean yield of 6.7 t ha^−1^ for the contemporarily used barley variety Sebastián.

## 1. Introduction

Climate change has become a major challenge in the 21st century [1,2] primarily due to its impact on the stability of global food security [3]. These changes in temperature, precipitation, and climate variability are introducing new challenges to agricultural output [4]. Climate change may lead to consequences that affect both the world as a whole and specific regions [5], and the impacts of climate change on yields have shown large spatial variations [6,7,8,9]. Long-term weather data are essential to evaluate the effects of climate change on crop production [7]. Weather and its variables, such as air temperature, precipitation patterns, and solar radiation, are major elements that determine the yield potential of crops [2,5,10,11,12], particularly impacting cereal crop yield [13].

Barley (*Hordeum vulgare* L.) is an ancient crop cultivated across different latitudinal zones around the world [7]. In Europe, barley is the second leading cereal crop for cool climates, after wheat [14]. Barley is considered one of the primary arable crops in the Czech Republic [15]; specifically, it is the third most cultivated crop, following wheat and rapeseed. In 2023, barley covered approximately 321 thousand ha (192 thousand of spring barley) of Czech Republic arable land, with a total grain yield of 1.8 million (950 thousand of spring barley) tons and average grain production of 5.5 t ha^−1^ (4.9 t ha^−1^ for spring barley) [16]. The primary use of barley in the Czech Republic is livestock feed, comprising nearly 70% of its production. The remaining portion is allocated to the brewing and distilling industries, which place greater emphasis on the quality of barley, particularly its protein content [17,18].

Temperature is one of the key environmental factors that influence the formation of spring barley yield [19]. According to Lobell et al. [20] and Xie et al. [21], barley is highly susceptible to global climate change, with rising temperatures causing a significant decline in global yields. Also, an increased variability of precipitation events can affect barley yield [5,6,9,14,22]. Compared to other crops, barley shows exceptional resilience, being able to adapt across diverse climatic zones and withstand harsh environmental challenges [23]. In addition to the direct effects of climate change on crop physiology, climate change indirectly influences yield and quality through changes in nutrient mineralisation and availability of crops [5].

To grow healthy, productive, and high-quality grain, it is essential to maintain balanced nutrition, which includes the right amounts of vital nutrients like nitrogen (N), [24], phosphorus [25], potassium [26], and sulphur [27]. All of these elements are crucial for crop development and growth, particularly under weather conditions marked by an increased frequency of extreme events. An imbalance in nutrients, whether a deficiency or an excess, can result in harmful conditions that interfere with the crop’s physiological processes. Consequently, this imbalance affects both the final yield and the quality of the produce. Among all macronutrients, N is the most important element as it is a fundamental part of amino acids and proteins, significantly influencing metabolic processes in plants. Application of this nutrient in the form of fertilisers significantly affects grain yield, its chemical composition and quality [27,28,29,30,31], and may act as a factor in increasing crop resistance against pathogenic organisms [32] and environmental stress conditions [24,33].

The application of too low N doses results in lower grain yields or grain quality that is inadequate for the intended use. Similarly, too high doses of mineral N can negatively affect both yield and quality parameters of barley grain. From a quality point of view, too high N doses are counterproductive for the distilling industry due to the high protein content of the grain [31,34]. In fact, barley increases the protein concentration of the grain as the N rate increases because barley plants use the available N even after the yield requirements have been met [35]. High doses of mineral N can also adversely affect grain yields. This may be due to lodging in the field [34], shortening of the root system [36], or altered metabolic processes in the plant [37]. At the same time, too high doses of mineral N are undesirable because of the potential negative impact on all segments of the environment [38,39], soil properties [40], and financial losses for farmers [41]. Finally, a well-chosen barley fertilisation strategy also influences yield stability, which is increasingly important for farmers [42].

Based on the outlined information, data from long-term experiments can assist in assessing how climate change affects crops and in explaining the effects of annual weather variability on yields [13,14,43]. This research aims to provide insights into the influence of weather and fertilisation on spring barley grain yield, utilising data from a field trial that has been ongoing in Prague since 1955. The primary objective was to investigate weather changes at the long-term trial location and discover potential relationships between these weather conditions and the yield of spring barley. Additionally, we analysed the development of barley grain yield as affected by different varieties utilised during the trial and how various fertilisation practices influenced barley grain yield, including the optimisation of N dosage for the modern barley variety, Sebastián. Finally, the influence of fertilisation on the inter-annual variability of yield was calculated.

## 2. Results

### 2.1. Temperature, Sunshine Duration, and Precipitation during Long-Term Field Trials

**Figure 1:**
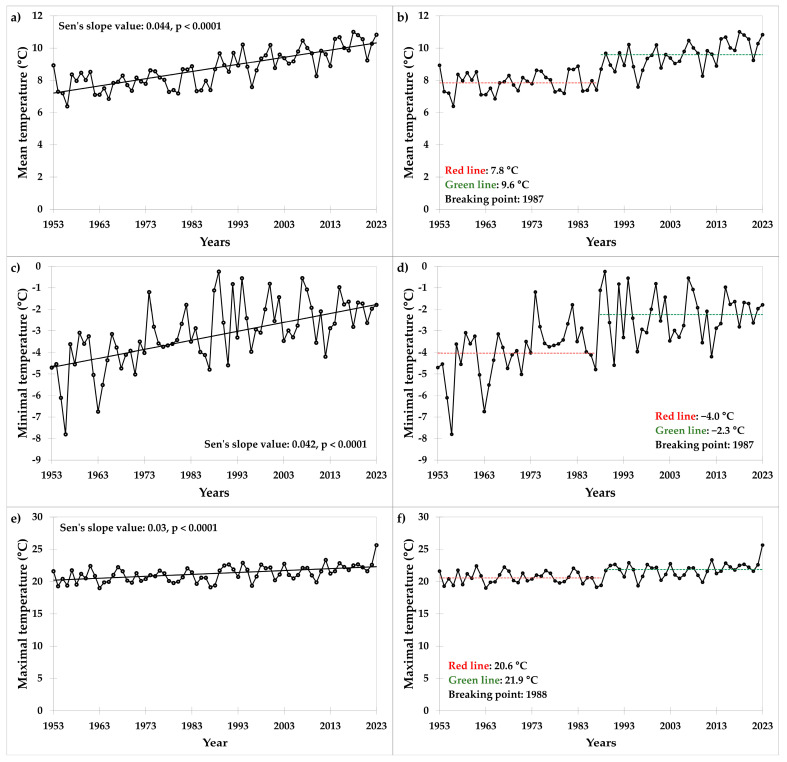
Values of temperature (°C): (**a**) mean, (**c**) minimal, and (**e**) maximal, and the Pettitt’s test for (**b**) mean, (**d**) minimal, and (**f**) maximal temperatures at the trial site in the period 1953–2023. Results of the Pettitt’s test (homogeneity test) showing two periods with different mean temperatures (red and green lines), and breaking points (year of change).

Since 1953, the mean, minimal, and maximal air temperatures have gradually increased at the site of a long-term field trial in Prague (Figure 1a,c,e). Temperature trends are increasing and statistically significant. The mean annual increases in mean, minimal, and maximal air temperatures are 0.044 °C, 0.042 °C, and 0.030 °C, respectively. Based on the homogeneity test (Pettitt’s test), 1987 and 1988 were identified as the breakthrough years, separating the period 1953–2023 into two periods with different mean, minimal, and maximal temperatures (Figure 1b,d,f).

**Figure 2:**
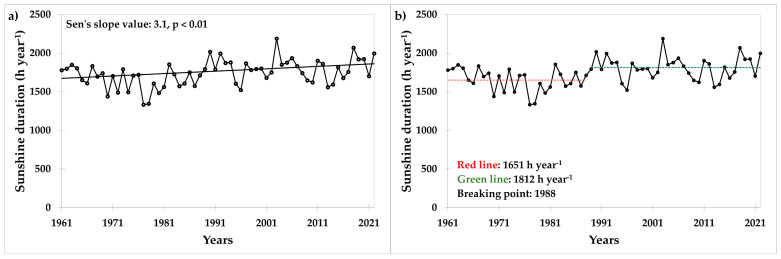
Values of (**a**) sunshine duration (h year^−1^) and (**b**) Pettitt’s test at the trial site in the period 1961–2022. Results of the Pettitt’s test (homogeneity test) showing two periods with different sunshine durations (red and green lines) and the breaking point (year of change).

The trend for sunshine duration from 1961 to 2022 was upward and statistically significant (Figure 2a); the average annual increase was 3.1 h, and the year 1988 emerged as the turning point (Figure 2b).

**Figure 3:**
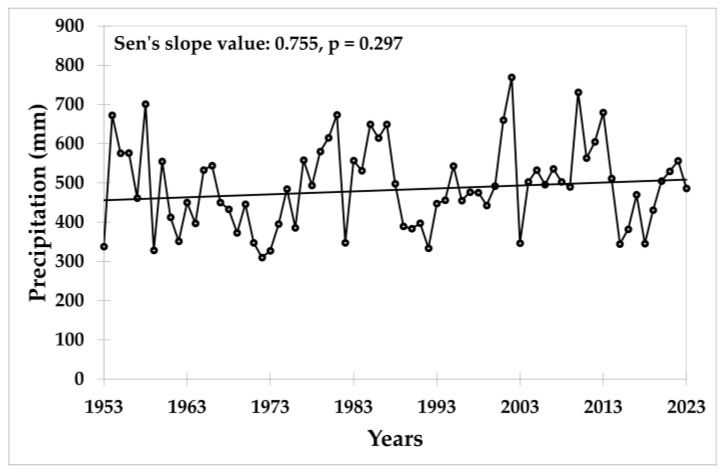
Values of precipitation at the trial site in the period 1953–2023.

With a Sen’s slope value of 0.755 mm, the precipitation trend was slightly upward but statistically insignificant (Figure 3).

**Figure 4:**
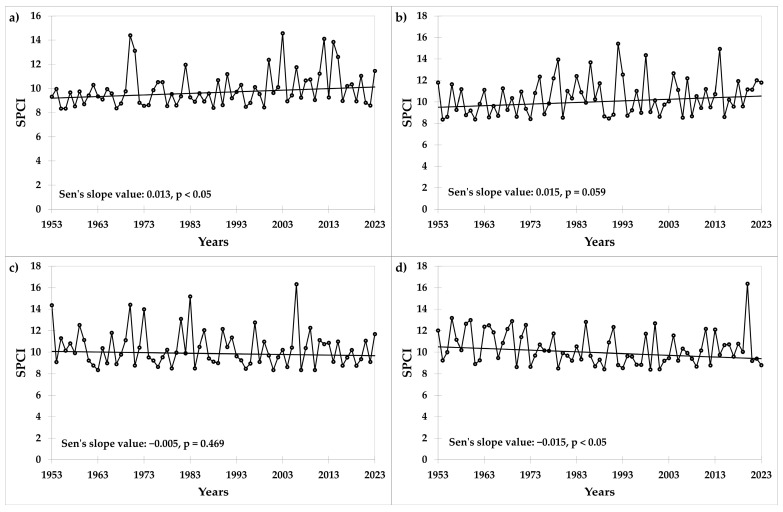
Values of the seasonal precipitation concentration index (SPCI) in the (**a**) first (I.–III.), (**b**) second (IV.–VI.), (**c**) third (VII.–IX.), and (**d**) fourth (X.–XII.) quarters of the year at the trial site in the period 1953–2023.

However, the distribution of precipitation is changing. The trends for the seasonal precipitation concentration index (SPCI) for the first quarter of the year (I.–III.) significantly increased (Figure 4a), and were on the edge of significance (*p* = 0.059) for the second quarter of the year (IV.–VI., Figure 4b), indicating a growing irregularity of precipitation during these periods. The breaking point for the first quarter occurred in 1999. In the third quarter (VII.–IX.), the SPCI values did not follow any trend (Figure 4c), while a downward trend was recorded for the last quarter of the year (*p* < 0.05), leading to more stable rainfall stratification (Figure 4d). The differences in SPCI values between the first (1953–1973) and last (2003–2023) twenty years of the trial were statistically significant (*p* < 0.05) for the first (I.–III.) and second (IV.–VI.) quarters of the year.

The correlation analysis confirmed strong and positive relationships between mean, minimal, and maximal temperatures and a moderate and positive relationship between mean and maximal temperatures and sunshine duration (Table 1). On the other hand, precipitation was weakly and negatively correlated with maximal temperature and duration of sunshine.

### 2.2. Grain Yield Development and the Effect of Fertilisation

According to the factorial ANOVA, the barley grain yield was influenced by the year (*p* < 0.001) and fertiliser treatment (*p* < 0.001) during the field trial. The impact of the two factors was comparable. While the “year” factor influenced yield by 48% (*df* = 43, *p* < 0.001), the fertiliser treatment influenced yield by 52% (*df* = 9, *p* < 0.001). The influence of the ‘year’ factor on yields indicates that barley yields were affected by the climatic conditions specific to each season. However, the term ‘year’ does not refer solely to the influence of the year itself. In fact, long-term trials adapt to their time, and different crop varieties were used throughout the trial. The assessment of the ‘year’ factor would be accurate only if the same variety of barley were used throughout the trial. Such a procedure is not possible because the trial would yield accurate but outdated results. The term ‘year’ must, therefore, be understood as the combined effect of both season and variety. Both factors have a definite impact on yield, but it is difficult to separate these effects from a statistical point of view. According to the experience of the crop breeder (personal communication), the influence of new varieties is dominant, as the significant impact of weather on crop yields is particularly pronounced in abnormal years. The results of the factorial ANOVA (comparing the fertiliser treatment and variety) support this claim, as the effect of fertiliser treatment on barley grain yield decreased to 17% (*df* = 9, *p* < 0.001), while the impact of variety accounted for 82% (*df =* 8, *p* < 0.001). The influence of variety on barley grain yield is shown in Table 2. The lowest grain yields were recorded in the two oldest varieties, Hanácký Kargyn and Branišovický, while the two latest varieties, Amulet and Sebastián, provided the highest yields.

**Figure 5:**
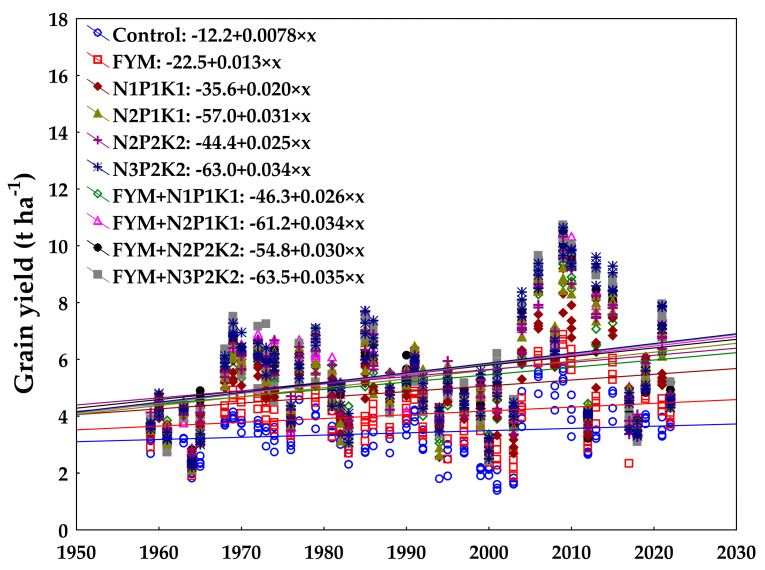
Development of grain spring barley yield (t ha^−1^) of individual fertiliser treatments since trial establishment (44 seasons).

Barley grain yields have been increasing across all fertiliser treatments since the trial was established (Figure 5). The lowest interannual yield increase was recorded for the unfertilised Control. The increasing trend for this treatment indicates the sustainability of growing barley under the given soil and climatic conditions, even without fertiliser application. As the amount of nutrients applied increased, the interannual yield increases were higher, with the N4P2K2, FYM+N2P1K1, and FYM+N4P2K2 treatments peaking.

Fertiliser treatment had a significant impact on the average grain yield of spring barley (*p* < 0.0001). Results are displayed in Table 3.

The lowest yields were noted in the unfertilised Control treatment (Table 3). Significantly higher yields were obtained in the FYM treatment (FYM applied to the preceding crop). The nutrients provided by the FYM through mineralisation thus contributed positively to a slight but significant increase in yield (+21%) when compared to the Control. The application of mineral fertilisers resulted in a further significant increase in yields. However, the differences between treatments (from N1P1K1 to FYM+N4P2K2) were not statistically significant. Although FYM contributed a certain amount of nutrients, this contribution did not have any effect when applied in conjunction with mineral fertilisers. Among the eight treatments compared, the lowest yields were consistently observed in treatment N1. This demonstrates the importance of N for yield production. On the other hand, the various doses of mineral P and K fertilisers had no effect on yields.

**Figure 6:**
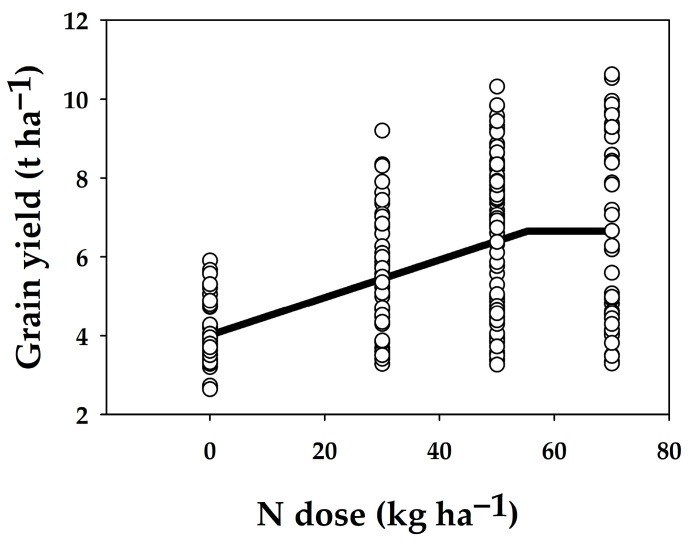
Values of Sebastián variety grain yields (t ha^−1^) in the period 2006–2022 (12 seasons). Yield data (circles) are interleaved with a linear–plateau model (black line).

The optimisation of mineral N fertilisation was determined based on the yield of the current barley variety, Sebastián, which has been used since 2008. Based on a linear–plateau response model (Figure 6), the optimal mineral N rate was set at 55 kg ha^−1^ N, corresponding to an average yield of 6.7 t ha^−1^. For the response model, only yields from the minerally fertilised treatments were used. Treatments with FYM were not included. Concerning the P and K fertilisers, P1 and K1 treatments provided comparable results as P2 and K2 treatments, and the slight difference between P2 and K2 treatments seems to be a function of increased N dose (difference between N1P1K1 and N2P1K1 treatments, Table 4).

### 2.3. Relationship between Grain Yield and Weather

Only 3 out of 220 analysed relationships between weather parameters and grain yield were found to be significant. These were (1) June precipitation (r = 0.4), (2) minimal temperature in July (r = 0.3), and (3) sunshine duration in May (r = −0.5).

**Figure 7:**
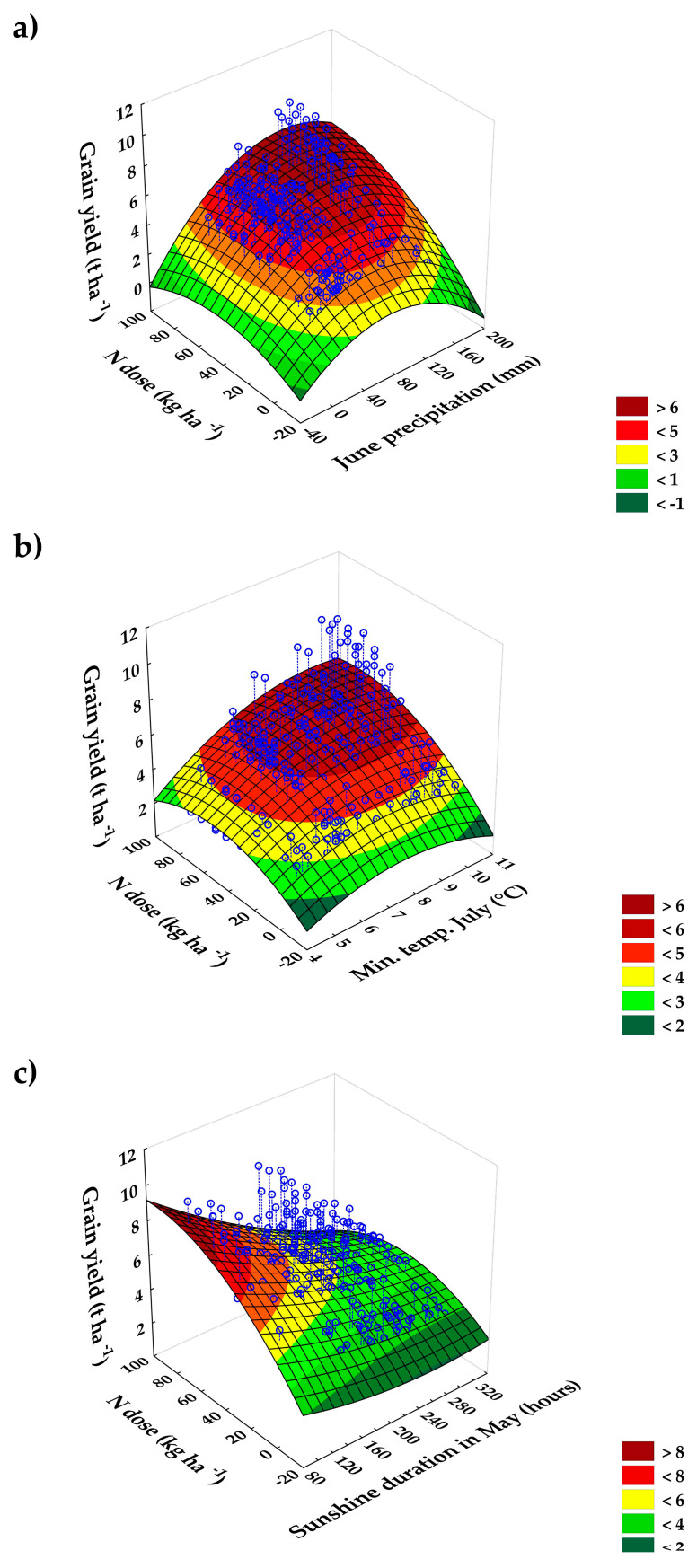
Relationships between grain yield (t ha^−1^), N dose (kg ha^−1^), and (**a**) June precipitation (mm), (**b**) minimal temperature in July (°C), and (**c**) sunshine duration in May (hours).

The relationship between June precipitation and grain yield is positive. Lower precipitation is associated with lower yields, and vice versa. The range of 100 to 120 mm represents the optimum level at which the highest yields can be expected. The effect is stronger with higher mineral N rates (Figure 7a). A similar relationship was observed between grain yield and minimal temperature in July. Higher minimal temperatures are associated with increased yields, and this effect is more pronounced when higher mineral N rates are applied (Figure 7b). On the other hand, the duration of sunshine in May is negatively correlated with yield (Figure 7c). At the same time, sunshine duration is significantly and negatively correlated with May rainfall (r = −0.6, *p* < 0.0001). Thus, sunny weather in May, which is associated with lower rainfall, is a negative factor for barley grain yield.

### 2.4. Yield Stability of Spring Barley

Based on the results of Kang’s rank-sum statistic, the most stable yields were provided by N2P2K2 treatment (rank 1), followed by N3P2K2, FYM+N1P1K1, FYM+N2P1K1, and FYM+N2P2K2 treatments (rank 2), N2P1K1 (rank 6), FYM+N3P2K2 (rank 7), N1P1K1 (rank 8), FYM (rank 9), and Control (rank 10).

The results show that without the support of externally supplied nutrients (Control), barley was significantly affected by external factors, soil–climatic conditions. The ability to withstand unfavourable conditions was diminished, while the capacity to exploit its potential under favourable conditions was restricted. This is reflected in low yields and high inter-annual variability in yields. Similarly, in the FYM treatment, the amount of nutrients available to barley was relatively low, uncertain, and unstable. The application of mineral fertilisers significantly reduced the inter-annual variability of yields. Mineral fertiliser acted as a stabilising element, resulting in higher yields. The crop was better able to withstand adverse conditions during poor seasons and, consequently, could maximise its yield potential in favourable conditions.

## 3. Discussion

### 3.1. Development of Weather at the Site of the Long-Term Trial

The primary factors driving agricultural production are temperature, precipitation, and their seasonal patterns [5,44]. At the trial site, all temperature trends increased and the relationship between temperature variables was confirmed. The results of this local change are similar to the findings of Mozny et al. [45], which indicated an increasing trend of the average annual air temperature in the Czech Republic from 1961 to 2020. Additionally, the study also noted an increase in the sum of effective temperatures above 5 and 10 °C during the vegetation period (April–September). Moreover, all temperature changes were more visible when datasets were divided into two periods: the past average period (1961–1990) and the current period (1991–2020). A similar trend was observed at the trial site, and results support current knowledge about the changing temperature trends in relation to global warming, which is well documented in Europe [46] and European countries, e.g., Germany [47], Lithuania [48], and Northern Serbia [49].

In the case of precipitation, which is the key element influencing water availability [50], the upward trend was not significant at the trial site. This is in accordance with the relatively stable amounts of rainfall in the Czech Republic [45]. Additionally, the number of days with soil drought increased between 1961 and 2020 [45], indicating a relationship with the changing seasonal distribution of precipitation. Similarly, in Europe, the frequency of drought events during spring and early summer indicated changes in the rainfall pattern [51]. In the context of global warming, investigating the temporal–spatial variability in precipitation using the precipitation concentration index (PCI) is important for making reliable predictions [52]. Therefore, the variability in precipitation conditions at the site of the long-term trial was evaluated by PCI on a seasonal scale—SPCI. This analysis revealed a significant trend of changing distribution in the first and fourth quarters.

Moreover, the separation of meteorological data into two twenty-year periods showed a significant trend in the first and the second quarters of the year. Both quarters showed a moderate, irregular concentration of precipitation values [52,53]. The trend of precipitation changes and its spatial variability has been observed in other research studies [52,54]. Therefore, rainfed barley production is at risk due to the anticipated reduction in precipitation and increases in mean temperature [2].

Besides temperature and precipitation, the duration of sunshine was also determined. The trend of sunshine duration significantly increased, with the turning point identified in the year 1988. The relationship between sunshine duration and mean and maximal temperature was confirmed. The results are in accordance with the report by [55] on climate variability in the Czech Republic between 1961 and 2020. Their study revealed an increase in the mean annual sunshine duration and seasonal sunshine duration (except for autumn) from 1991 to 2020 compared to the period from 1961 to 1990.

### 3.2. Influence of Weather on Grain Yield during the Long-Term Trial

Long-term weather records contain data from years with contrasting patterns of temperature and rainfall that can occur in the short term, particularly during a growing season. These records can serve as a proxy for assessing the potential effects of climate on spring barley [7]. A significant role of temperature in barley germination and the development of reproductive parts that contribute to yield has been previously reported [2,56,57,58]. The temperature outside the optimum range, especially an increase in temperature, affects the growing period by influencing photosynthesis and causing a shortening of the growing period, resulting in lower yields [5,19,59]. Despite the increase in temperature trends at the long-term trial site, spring barley’s grain yield was not affected. According to the research conducted by Yigit et al. [2], the daily peak temperature is the most critical factor influencing the development of spring barley yield.

It also affects soil water content through its effect on evaporation rates. In the long-term trial evaluation, precipitation and sunshine duration also significantly influence the grain yield of spring barley, indicating that sunny weather is associated with lower precipitation. Similar results were obtained for wheat in Japan [60]. An increase in mean daily sunshine hours after sowing led to a rise in wheat grain yield; however, this positive effect was negated by higher precipitation (>30 mm) during the early seedling stage [60]. Additionally, Trnka et al. [61] showed a reduction in spring barley yield in response to the seasonal meteorological drought in the Czech Republic (1961–2000).

Among the analysed relationships between individual weather parameters and the grain yield of spring barley, only three were found to be significant. These results can be attributed to the previously mentioned fact that barley, in comparison with other crops, has the greatest range of adaptation to varying climatic conditions [23]. At the site of the long-term trial, the relationship between grain yield and June precipitation, minimal temperature in July, and duration of sunshine in May confirmed that barley is moderately sensitive to soil water deficit and heat stress, which reduce tillering in early phenological stages [62]. Similarly, Thai et al. [14] reported the relationship between weather conditions during the early growing stages and the crop yield of spring barley. They specifically noted a negative effect of high average temperature in April, high temperature on the sowing day, and high precipitation in March. The latter can lead to a decrease in yield by leaching, which may reduce nutrient availability and by delaying sowing due to soil saturation [14,63]. Daničić et al. [49] also highlighted that extreme weather conditions during the growing season have a substantial effect on spring barley cultivation, with crop yields being closely tied to the availability and distribution of precipitation.

### 3.3. Influence of Fertilization and Yield Stability of Spring Barley

The lowest mean grain yields and yield stability were observed in the unfertilised Control and FYM treatments. Without any external nutrient supply, the plants in the unfertilised Control treatment group rely on nutrients from the soil that have not been fertilised since the trial was established in 1955. Nevertheless, the yields of the Control tended to increase, especially with the introduction of the modern Sebastián variety in 2006, which led to a noticeable increase in yield. This demonstrates the sustainability of spring barley production even without fertilisation. This development is likely attributed to the beneficial crop rotation that includes alfalfa (*Medicago sativa* L., two representations in a nine-year cycle), which is able to fix atmospheric N, providing this crucial nutrient to following crops, and has a beneficial effect on soil properties [64,65,66,67]. Similar results of spring barley cultivation were reported for faba bean (*Vicia faba*) pre-cultivation [68].

Application of FYM to the preceding sugar beet crop (*Beta vulgaris*) significantly increased barley grain yield compared to the unfertilised Control. The FYM is a valuable fertiliser used for maintaining soil fertility and improving plant nutrition, perhaps since the time of Europe’s first farmers eight thousand years ago [69]. Its application is associated with higher yields and soil organic carbon content [70], which beneficially influences soil biochemical properties [71] and nutrient availability [72]. Due to its high C/N ratio, FYM releases its nutrients through the mineralisation process more slowly than animal slurries, but over a longer period [73]. As a result, the FYM can positively influence the yields of wheat, maise, and rapeseed even three years after its application in the field [74,75]. However, the disadvantage of manure fertilisers is their heterogeneity. Manure composition depends on many factors, such as the type of animal and the technology used for animal housing [76,77]. Additionally, manure mineralisation significantly relies on soil and climatic conditions.

Mineral fertilisers, on the other hand, are homogeneous, have a defined composition, and produce a very rapid effect. In the long-term trial evaluation, no significant differences in barley grain yield were observed among all NPK treatments, regardless of FYM co-application. This is because barley has a very good N uptake and utilisation efficiency. In comparison with wheat, both cereals respond equally to N fertilisation. But due to lower uptake and utilisation efficiency, wheat requires high N doses to achieve optimal yields [78]. In other words, barley is able to utilise applied N fertilisers more efficiently, showing the highest N recovery rate, followed by maise, wheat, and rice [79]. Therefore, the application of high rates of N fertilisers is unnecessary. In the long-term trial evaluation, the optimal mineral N rate was determined to be 55 kg ha^−1^ N, which corresponds to an average yield of 6.7 t ha^−1^. This recommendation is valid for the given soil and climatic conditions and the modern barley variety (Sebastián). Applying higher doses would not be cost-effective and could have a negative impact on the environment [38,39,40,41,80]. Different N doses resulted in optimal outcomes under various soil–climate conditions and for different barley utilisations, ranging from 30 kg ha^−1^ N [29], 75 kg ha^−1^ N [81], 100 kg ha^−1^ [82] to 120–140 kg ha^−1^ [83].

Yield stability is a parameter that is gaining importance among farmers due to climate change [42]. Weather fluctuations introduce an increasing degree of uncertainty into crop production in terms of future and expected yields. Various methods can be used to reduce uncertainty, stabilise yields, and decrease inter-annual variability. These practices include crop rotation, fertilisation, and growing mixed varieties in the field simultaneously [84,85]. According to several studies, more stable yields of cereals are provided by N doses from the middle spectrum of dosages, especially when applied with manures [85,86]. Thus, richer crop rotations and the application of moderate doses of mineral nutrients (neither too high nor too low) lead to more stable yields. Mineral nutrient doses that are too low do not meet the needs of the crop, preventing the production of healthy and resilient crops. This makes them more vulnerable to weather fluctuations and pathogens.

Similarly, excessive mineral nutrient doses can increase the inter-annual variability of yields. This can occur due to higher lodging rates or as a result of morphological and metabolic changes in the crops [34,36,37]. In the long-term trial, N2P2K2 emerged as the treatment that offered the highest level of stability. The N3P2K2 treatment and the combination of manure with low mineral nutrient rates came in second. Conversely, the least stable production was observed for the low and high nutrient rates. Therefore, results of spring barley confirm that moderate fertilisation rates offer the highest level of stability under specific soil and climate conditions.

## 4. Materials and Methods

### 4.1. Field Trial Description

The long-running field experiment in Prague was initiated in 1955. The purpose of the experiment is to investigate the effects of diverse fertilisation treatments and climatic factors on crop productivity. The trial is situated in the immediate vicinity of the international airport in Prague (Václav Havel Airport Prague). Its coordinates are 50°05′15″ N, 14°17′28″ E. According to the Köppen–Geiger climate classification, the area is categorised as DfB [87]. The elevation of the field trial is 370 m above sea level. The long-term experiment mean, minimal, and maximal annual temperatures are 8.7 °C, −3.1 °C, and 21.2 °C, respectively (1953–2023). The mean annual total precipitation was 491.4 mm (1953–2023). The mean annual sunshine duration was 1740 h (1961–2022). The type of soil is orthic Luvisol [88], formed by diluvial sediments mixed with loess. The depth of the topsoil is around 0.3 m.

The long-term field experiment described and analysed in this article consists of three fields placed next to each other. The size of one experiment site is 144 × 96 m. Each site is divided into 96 experimental plots, with each plot measuring 12 × 12 m. In each field, 24 different fertilisation treatments are analysed, with four replications of each treatment (24 × 4 = 96 plots). For the purpose of this article, we evaluated a total of 10 fertilisation treatments: (1) Control (unfertilised since the trial establishment), (2) mineral nitrogen, phosphorus (P), and potassium (K)(N1P1K1), (3) N2P1K1, (4) N2P2K2, (5) N3P2K2, (6) cattle farmyard manure (FYM), (7) FYM+N1P1K1, (8) FYM+N2P1K1, (9) FYM+N2P2K2, (10) FYM+N3P2K2. The doses of mineral nutrients (kg ha^−1^) applied in the individual treatments are shown in Table 4.

Mineral N was used as lime ammonium nitrate, mineral P as superphosphate, and mineral K as potassium chloride. All mineral nutrients were applied in a single dose and spread manually by hand. While minerals P and K were incorporated in the autumn, mineral N was incorporated in the spring before barley was sown. To eliminate the edge effect, only the central area of the plot (5 × 5 m) was used for experimental purposes, specifically for grain and straw yield analysis. The cattle farmyard manure (FYM) was applied to the preceding crop, which was sugar beet, at a dose of 21 t ha^−1^. The average concentrations of N, P, and K in the FYM treatments were 5, 1.4, and 5.9 t ha^−1^, respectively, with the assumption that 8% of the initial N [80] was available to barley (8.4 kg ha^−1^).

A total of nine barley varieties were used during the experiment: (1) Hanácký Kargyn (1959–1961, 3 seasons), (2) Branišovický (1963–1965, 3 seasons), (3) Diamant (1968–1977, 8 seasons), (4) Favorit (1979–1983, 4 seasons), (5) Bonus (1985–1991, 5 seasons), (6) Perun (1992, 1 season), (7) Akcent (1994–2004, 7 seasons), (8) Amulet (2004, 1 season), and (9) Sebastián (2006–2022, 12 seasons). Barley was sown in early spring and harvested in July.

### 4.2. Data Analysis

The weather data come from meteorological stations located at the Crop Research Institute in Prague, operated by the Crop Research Institute, and at the Václav Havel International Airport in Prague, operated by the Czech Hydrometeorological Institute. The trends for mean, minimum, and maximum temperatures, sunshine duration, and precipitation were evaluated using the Mann–Kendall trend test [89,90], accompanied by Sen’s slope estimation [91]. The breaking point, which separates two time series with different mean values (temperature, sunshine duration, SPCI), was determined using Pettitt’s test [92]. The Seasonal Precipitation Concentration Index (SPCI) was calculated according to Equation (1), as proposed by [93]:

(1) $$SPCI\frac{\sum_{i=1}^{3}p_{i}^{2}}{{\left(\sum_{i=1}^{3}p_{i}\right)}^{2}}\times 25$$

where pi refers to the monthly precipitation in month i, measured each year throughout the observation period. The periods in our case represent four quarters, precisely January–March (I.–III.), April–June (IV.–VI.), July–September (VII.–IX.), and October–December (X.–XII.).

The grain yields were analysed by examining their distribution [94,95]. In the case of normally distributed data, Levene’s test was used to assess the equality of variances, followed by analysis of variance (ANOVA) and Tukey’s Honestly Significant Difference (HSD) test. If homogeneity of variance was violated, the Games–Howell test was employed. In the case of non-normally distributed data, the Kruskal–Wallis ANOVA was used [96], followed by the Conover–Iman procedure [97]. The factorial ANOVA was used to analyse the effect of fertilisation and year (variety) on grain yield. The relationships between weather parameters and grain yield were analysed using the Pearson (r) or Spearman (r_s_) correlation tests. The yield stability was analysed by Kang’s rank-sum statistic [98], using StabilitySoft [99]. For the optimisation of mineral nitrogen fertiliser, a linear–plateau response model was used.

Statistical analyses were conducted and graphical outputs generated using Statistica 14.0 (Tibco Software, Palo Alto, CA, USA), SigmaPlot 14.5 (Systat Software Inc., San Jose, CA, USA), and XLStat software (Lumivero, Burlington, MA, USA).

## 5. Conclusions

Barley grain yield is greatly impacted by key factors such as weather conditions, fertilisation, and the choice of barley variety. This study examines a long-term field trial initiated in Prague in 1955. The research analysed 44 growing seasons where barley was planted after sugar beet in the rotational cropping system. The study analysed temperature, precipitation, and sunshine duration at the trial site, with correlation analysis used to determine the relationships between weather conditions and grain yield outcomes. In terms of fertilisation, ten different treatments were analysed, including the optimisation of nitrogen (N) doses using the linear–plateau model. Additionally, the effect of these treatments on grain yield stability was evaluated. The conclusions are as follows:

(i). Trends for mean, minimal and maximal temperatures are on the rise, confirming a warming trend. The duration of sunshine is also increasing. While annual precipitation does not show significant changes at the long-term experiment site, the distribution of precipitation is gradually shifting towards greater irregularity in the first and second quarters of the year. Factors such as June rainfall, minimum temperature in July, and the length of sunshine in May significantly influence barley grain yields.
(ii). The application of manure to the preceding crop positively affects barley grain yield. Mineral fertiliser application results in the highest yields. The linear–plateau model indicates that applying 55 kg ha^−1^ of nitrogen produces an optimal yield of 6.7 t ha^−1^.
(iii). Moderate fertilisation was most stable for grain production, while the unfertilised Control, farmyard manure (FYM), and the highest mineral fertiliser doses exhibited more significant inter-annual yield fluctuations.

## Figures and Tables

**Table 1:** Correlation coefficients showing relationships between mean, minimal and maximal temperatures, sunshine duration, and precipitation in the period 1953–2023. Bold values are statistically significant (*p* < 0.05).

	Mean Temp.	Minimal Temp.	Maximal Temp.	Sunshine Duration	Precipitation
Mean temp.	1	**0.733**	**0.735**	**0.544**	−0.081
Minimal temp.	**0.733**	1	**0.466**	0.201	0.002
Maximal temp.	**0.735**	**0.466**	1	**0.547**	**−0.273**
Sunshine duration	**0.544**	0.201	**0.547**	1	**−0.337**
Precipitation	−0.081	0.002	**−0.273**	**−0.337**	1

**Table 2:** Grain yield (t ha^−1^) of individual spring barley varieties in the period 1953–2023. Mean data represent average values ± standard deviations. Different letters in the mean column indicate significant differences (*p* < 0.05, Conover–Iman test) among varieties. No statistically significant variation exists between values assigned the same letter.

Variety	Mean	Minimum	Maximum
Hanácký Kargyn	3.7 ± 0.5 ^AB^	2.7	4.8
Branišovický	3.3 ± 0.9 ^A^	1.8	4.9
Diamant	5.4 ± 1.1 ^E^	2.7	7.5
Favorit	4.6 ± 1.1 ^D^	2.3	7.1
Bonus	5.3 ± 1.1 ^E^	2.7	7.7
Perun	4.4 ± 0.7 ^CD^	2.8	5.7
Akcent	4.0 ± 1.0 ^BC^	1.4	6.2
Amulet	6.5 ± 1.3 ^F^	3.6	8.4
Sebastián	6.1 ± 2.1 ^EF^	2.3	10.7

**Table 3:** Spring barley grain yields (t ha^−1^) for individual fertiliser treatments over the analysed period (44 seasons). Mean data represent average values ± standard deviations. Different letters in the mean column indicate significant differences (*p* < 0.05, Conover–Iman test) among fertiliser treatments. No statistically significant variation exists between values assigned the same letter.

	Mean	Minimum	Maximum
Control	3.4 ± 0.9 ^A^	1.4	5.9
FYM	4.1 ± 1.0 ^B^	1.9	6.9
N1P1K1	4.9 ± 1.3 ^C^	2.2	9.2
N2P1K1	5.3 ± 1.6 ^C^	2.1	9.3
N2P2K2	5.4 ± 1.6 ^C^	2.3	10.3
N3P2K2	5.5 ± 1.9 ^C^	2.2	10.6
FYM+N1P1K1	5.2 ± 1.5 ^C^	2.3	9.7
FYM+N2P1K1	5.5 ± 1.8 ^C^	2.2	10.3
FYM+N2P2K2	5.5 ± 1.8 ^C^	2.2	10.5
FYM+N3P2K2	5.5 ± 1.9 ^C^	2.2	10.7

**Table 4:** Application of mineral nutrients (N, P, K, kg ha^−1^) in the individual fertiliser treatments. Doses indicate the amount of net nutrients applied.

Treatment	N (kg ha^−1^)	P (kg ha^−1^)	K (kg ha^−1^)
Control	0	0	0
N1P1K1	30	21.1	66.4
N2P1K1	50	21.1	66.4
N2P2K2	50	26.4	83
N3P2K2	70	26.4	83

## Data Availability

Dataset available on request from the authors.
